# Systematic Review of Autism in Women: Invisibilised Profiles and Gender Bias

**DOI:** 10.31083/RN49367

**Published:** 2026-04-23

**Authors:** Ana Muiño Tato

**Affiliations:** ^1^Department of Psychology, Faculty of Health Sciences, Universidad UNIE, 28046 Madrid, Spain

**Keywords:** autism spectrum disorder, diagnostic bias, sex factors, mental health services

## Abstract

**Introduction:**

Autism spectrum disorder (ASD) shows marked clinical heterogeneity and a pronounced sex disparity in diagnosis, with male-to-female ratios of up to 4.6:1 in the Spanish educational context, suggesting potential systematic under-identification in females. This systematic review critically examines gender bias in ASD diagnosis, focusing on females, and assesses whether current diagnostic criteria adequately capture Level 1 presentations.

**Method:**

A literature search was conducted in December 2025 across American Psychological Association’s database (APA) PsycInfo, Dialnet, PsicoDoc and Education Resources Information Center (ERIC), using Population, Exposure, Comparator, Outcome (PECO) and Patient, Intervention, Comparison, Outcome (PICO) frameworks. Following the Preferred Reporting Items for Systematic Reviews and Meta-Analyses (PRISMA) process, 26 studies (2015–2025) were included, encompassing observational, psychometric, technological, quasi-experimental and qualitative designs. Overall methodological quality was good to very good (mean CRF-QS = 15.7), and risk of bias was predominantly low.

**Results:**

Findings indicate consistent diagnostic inequities: delayed diagnosis in girls, reduced sensitivity of standard instruments to less externalizing phenotypes, the role of social camouflaging, and moderating effects of comorbidities and contextual factors.

**Conclusion:**

Diagnostic bias appears multifactorial and calls for gender-sensitive, multi-method and intersectional approaches to ensure equitable access to diagnosis and support.

## 1. Introduction

Autism spectrum disorder (ASD) is a neurodevelopmental condition characterised by persistent difficulties in social communication and interaction, alongside restricted and repetitive patterns of behaviour, interests, or activities [1]. Autism is a highly heterogeneous condition, and this variability is particularly pronounced in women, whose clinical presentation often diverges from the traditionally described male-based phenotype.

Over recent decades, the estimated prevalence of ASD has increased steadily. This rise has been attributed, at least in part, to greater societal awareness, the broadening of diagnostic criteria, and improvements in early identification and diagnostic practices [2].

In Spain, available epidemiological data—primarily derived from the educational system—indicate that 64,198 boys (82.24%) and 13,865 girls (17.76%) with ASD are enrolled in non-university education, representing approximately 0.94% of the total student population [2]. This distribution reflects a ratio of approximately 4.6 boys for every girl diagnosed, which is consistent with international estimates reporting male-to-female ratios ranging from 3:1 to 5:1 [3].

The interpretation of these figures reveals a marked sex disparity. This systematic under-identification of ASD in girls and women has often been interpreted as reflecting a true difference in prevalence [4]. However, accumulating evidence suggests that autistic girls and women frequently present with less prototypical phenotypic profiles, characterised by fewer externalising behaviours, subtler social communication difficulties, and greater apparent competence in structured social contexts. These features can hinder detection when standard diagnostic procedures are applied [5]. Moreover, social camouflaging strategies—defined as conscious or implicit attempts to mask or compensate for core autistic difficulties in order to conform to normative social expectations—have been consistently documented in females on the autism spectrum [6].

Difficulties in the accurate identification of autism in females contribute to delayed diagnosis, underdiagnosis, and more complex clinical trajectories, as well as an increased risk of mental health difficulties during adolescence and adulthood. Despite this, most diagnostic frameworks and screening instruments currently in use have been developed and validated predominantly in male samples. This raises critical concerns regarding their sensitivity, specificity, and overall validity for adequately capturing the phenotypic diversity of ASD in girls and women.

Against this background, the aim of the present systematic review is to critically examine the existing literature on gender bias in the diagnosis of ASD, with a particular focus on women and individuals assigned female at birth. The review seeks to evaluate the extent to which current diagnostic criteria and assessment tools adequately reflect female autism phenotypes. Specifically, the objectives are: (1) to describe recent evidence on sex- and gender-related diagnostic disparities and their variation across contexts and data sources; (2) to analyse whether diagnostic delays occur more frequently in girls and women, and to identify associated factors such as comorbidities (e.g., Attention-Deficit/Hyperactivity Disorder) and contextual influences; (3) to examine the performance of screening and diagnostic instruments (e.g., Social Communication Questionnaire (SCQ), Autism Diagnostic Observation Schedule, Second Edition (ADOS-2), Modified Checklist for Autism in Toddlers, Revised (M-CHAT-R), with particular attention to potential gender biases in sensitivity, specificity, and cut-off thresholds); (4) to synthesise available psychometric evidence regarding factor structure and measurement invariance of key instruments in female samples; and (5) to integrate qualitative findings on social camouflaging, gender stereotypes, and systemic barriers, and to discuss their implications for clinical practice, diagnostic processes, and future research.

## 2. Method

This work is based on a systematic review of the scientific literature, the primary objective of which is to analyse gender bias in the diagnosis of ASD. Specifically, it aims to examine the diagnostic difficulties affecting women on the autism spectrum, with particular attention to factors such as social camouflaging, a lower prevalence of externalising symptoms, and clinical profiles that diverge from traditional, male-normative diagnostic models.

A systematic review was selected as the most appropriate methodological approach given that gender bias in ASD diagnosis has been investigated using highly heterogeneous study designs—including observational, psychometric, qualitative, and technological approaches—and has addressed a wide range of outcomes, such as age at diagnosis, diagnostic instrument performance, subjective diagnostic experiences, and structural or systemic barriers to care. In this context, a systematic review enables the identification of convergent patterns across methodologies, facilitates comparisons between different analytical approaches, and allows for the delineation of key gaps in the existing evidence base. Compared with descriptive or primary exploratory designs, this approach is better aligned with the objective of critically synthesising the available literature and assessing the extent to which current diagnostic models and tools adequately capture female or otherwise less prototypical autism profiles.

Using the population, intervention, comparison, and outcome (PICO) framework, the following research question was formulated to guide the review: Do the current diagnostic and statistical manual of mental disorders, fifth edition, text revision (DSM-5-TR) diagnostic criteria adequately represent women with ASD Level 1?

### 2.1 Inclusion and Exclusion Criteria

Rigorous inclusion and exclusion criteria were established to ensure methodological consistency and scientific quality across the studies included in the review.

#### 2.1.1 Type of Studies 

The following study designs were included: (1) Observational studies (cross-sectional, cohort, retrospective, and population surveillance studies). (2) Psychometric or methodological studies (e.g., factor structure, measurement invariance, validity, and screening accuracy). (3) Experimental or technological studies applied to identification or diagnosis (e.g., artificial intelligence-based models, eye-tracking paradigms, point-of-view or wearable technologies). (4) Qualitative studies focusing on diagnostic trajectories, self-diagnosis, gender stereotypes, and gender socialisation processes. (5) Quasi-experimental studies aimed at improving diagnostic services or pathways (e.g., pre–post service evaluations). The following were excluded: (1) Reviews (systematic or narrative), editorials, commentaries, letters, and study protocols without empirical data. (2) Studies that did not report specific outcomes related to diagnosis, age at diagnosis, diagnostic instrument performance, diagnostic inequalities, or diagnostic trajectories.

#### 2.1.2 Publication Date

Studies published between 2015 and 2025 were included. This period was selected to capture recent changes in diagnostic conceptualisation, assessment tools, clinical practice, and intersectional approaches to ASD, while also encompassing earlier large-scale epidemiological or surveillance studies that provide a relevant contextual foundation.

#### 2.1.3 Language

Studies published in English or Spanish were included in order to maximise accessibility and minimise linguistic bias.

#### 2.1.4 Population

The following populations were included: (1) Individuals with a confirmed ASD diagnosis or those referred for ASD assessment. (2) General population samples when autistic traits or screening outcomes were assessed using standardised instruments. (3) Children and adolescents (0–18 years) as well as adults, when the study objective focused on diagnostic experiences, self-diagnosis, or gender-related diagnostic bias. (4) Studies reporting analyses by sex assigned at birth, gender identity, race/ethnicity, and/or socioeconomic status. (5) Women with a confirmed ASD diagnosis, without age restriction, diagnosed according to diagnostic and statistical manual of mental disorders, fifth edition (DSM-5) or DSM-5-TR criteria. (6) Studies including self-diagnosed or self-identified autistic individuals were included only when examining diagnostic trajectories, barriers to access, or lived experiences related to gender bias; these studies were analysed separately within the qualitative synthesis.

The following were excluded: Studies focusing exclusively on clinical, educational, or therapeutic interventions unrelated to identification, screening, diagnosis, or diagnostic inequalities.

#### 2.1.5 Exposure/Intervention

Included exposures and diagnostic-related factors were: (1) Sex and gender (e.g., AMAB (Assigned Male At Birth) and AFAB (Assigned Female At Birth); girls/boys; women and gender-diverse individuals). (2) Race/ethnicity and socioeconomic status. (3) Comorbidities or prior diagnoses potentially influencing diagnostic pathways (e.g., attention-deficit/hyperactivity disorder). (4) Social camouflaging or masking behaviours, assessed quantitatively or explored qualitatively. (5) Use or evaluation of diagnostic and screening instruments (e.g., SCQ, ADOS-2, CAT-Q - Camouflaging Autistic Traits Questionnaire, M-CHAT-R). (6) Artificial intelligence-based models or digital/ecological assessment tools (e.g., Canvas Dx, OpenFace/MediaPipe, machine learning applied to parental reports or behavioural data). (7) Interventions aimed at improving diagnostic services or pathways.

#### 2.1.6 Comparison

Eligible comparisons included: (1) Comparisons between sexes or genders. (2) Comparisons across racial or ethnic groups. (3) Comparisons across socioeconomic strata. (4) Individuals with versus without an ASD diagnosis. (5) Individuals with versus without a prior ADHD diagnosis. (6) Pre–post comparisons in studies evaluating service improvements. (7) Thematic comparisons within qualitative studies.

#### 2.1.7 Outcomes

Studies were included if they reported at least one of the following outcomes: (1) Age at diagnosis, diagnostic delay, or waiting times. (2) Prevalence estimates and demographic differences. (3) Probability of receiving an ASD diagnosis and associated predictors. (4) Screening or diagnostic performance indicators (e.g., sensitivity, specificity, accuracy, detection rates). (5) Psychometric properties of instruments (e.g., factorial structure, measurement invariance). (6) Access to and continuity of diagnostic or clinical care. (7) Qualitative accounts of bias, gender stereotypes, self-diagnosis, or barriers to diagnosis. (8) Impact of diagnostic timing on mental health outcomes, including anxiety and depression.

### 2.2 Search for Studies

The bibliographic search was conducted in December 2025 using electronic databases considered highly relevant to the fields of clinical psychology, mental health, education, and therapeutic intervention: APA PsycInfo (https://www.apa.org/pubs/databases/psycinfo), Dialnet (https://dialnet.unirioja.es/), PsicoDoc (https://www.psicodoc.org/), and ERIC (https://eric.ed.gov/). These databases were selected on the basis of disciplinary relevance, thematic coverage, and institutional accessibility, with particular emphasis on sources offering robust representation of research in autism diagnosis, child and adolescent mental health, educational psychology, and gender studies.

APA PsycInfo and ERIC were prioritised due to their comprehensive coverage of empirical and theoretical literature on ASD assessment and diagnostic processes. Dialnet and PsicoDoc were included to facilitate access to Spanish-language publications, thereby reducing linguistic bias and incorporating evidence from Ibero-American contexts that is often underrepresented in international reviews.

**Fig. 1.**
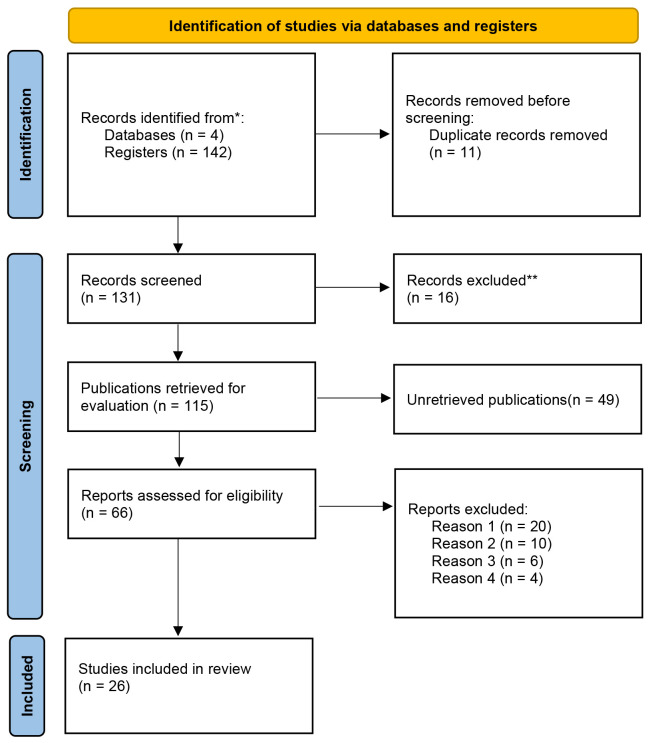
**Flow diagram**. *Consider, if feasible to do so, reporting the number of records identified from each database or register searched (rather than the total number across all databases/registers). **If automation tools were used, indicate how many records were excluded by a human and how many were excluded by automation tools.

Multidisciplinary databases such as Scopus and Web of Science were not included. This decision may have limited the retrieval of certain international studies indexed exclusively in those platforms; however, it was based on accessibility considerations and on preliminary scoping indicating a substantial overlap of key studies within the databases selected. This limitation is explicitly acknowledged and considered when interpreting the generalisability of the findings, particularly with regard to geographical regions and academic disciplines less represented in the consulted databases. The review was conducted and reported in accordance with the preferred reporting items for systematic reviews and meta-analyses (PRISMA) reporting standards. The processes of study identification, screening, eligibility assessment, and inclusion were reported in accordance with the PRISMA guidelines (see **Supplementary Material**), including the corresponding flow diagram (Fig. 1). Given the aim of this review—to integrate evidence derived from heterogeneous methodologies (quantitative, qualitative, and technological)—the synthesis prioritised a structured narrative approach, alongside an explicit appraisal of methodological quality and risk of bias.

As this review was conducted within the framework of an academic research project and was not prospectively registered, certain formal PRISMA components (e.g., protocol registration) were not implemented. This is acknowledged as a methodological limitation. Nevertheless, the core principles of the PRISMA framework were adhered to, including a reproducible search strategy, clearly defined inclusion and exclusion criteria, a staged selection process, and transparent reporting of study flow.

Reasons for study exclusion were grouped by category to prevent duplication across screening phases (title/abstract screening versus full-text assessment), as illustrated in the PRISMA flow diagram.

Table 1 presents the detailed search strategy, including Boolean operators (AND/OR) and key search terms used across databases.

**Table 1.** **Search strategy (Boolean AND/OR)**.

Patients	Interventions/Exposures	Comparison	Outcomes
autism OR autistic OR “autism spectrum disorder” OR ASD OR neurodivergent OR “autism traits” OR “autism-associated traits	diagnos OR “age at diagnosis” OR “diagnostic delay” OR “diagnostic pathway” OR identification OR detection OR screening OR assessment	male OR female OR boys OR girls OR AFAB OR AMAB	prevalence OR epidemiology OR “age of diagnosis” OR “time to diagnosis” OR “waiting time”
	OR “camouflaging” OR masking OR compensation OR assimilation	OR “racial” OR ethnic OR “non-Hispanic” OR Hispanic	OR sensitivity OR specificity OR accuracy OR “positive predictive value” OR “negative predictive value” OR PPV OR NPV
	OR “sex differences” OR sex OR gender OR female OR women OR girls OR AFAB OR AMAB OR nonbinary OR transgender OR “gender diverse”	OR SES OR poverty OR “income”	OR psychometric OR “factor structure” OR invariance OR “measurement invariance” OR validity OR reliability
	OR race OR ethnicity OR “socioeconomic status” OR SES OR poverty OR deprivation		OR “lived experience” OR qualitative OR interview OR “thematic analysis” OR phenomenolog
	OR ADHD OR “prior diagnosis”		

Note. Terms per block to combine.

Given that the objective of this review was to analyse gender bias in the diagnosis of ASD from a broad and integrative perspective—including structural inequalities, diagnostic trajectories, and the performance of diagnostic tools—a combined methodological framework was adopted. Specifically, both the population, exposure, comparison, and outcome (PECO) and PICO frameworks were employed to ensure alignment between the research questions and the heterogeneous designs of the included studies.

The PECO framework, which is particularly suited to observational and epidemiological research, was used to capture diagnostic inequalities associated with sex/gender, race/ethnicity, socioeconomic status, and other contextual or systemic factors. Complementarily, the PICO framework was applied to studies focusing on screening instruments, diagnostic assessments, or technological approaches, where the comparison between interventions, reference standards, and diagnostic accuracy outcomes is methodologically more appropriate. This dual-framework strategy enabled a coherent integration of evidence across diverse methodological approaches while maintaining consistency with the overarching objectives of the review.

### 2.3 Inclusion Criteria

Main PECO Framework (Diagnostic Inequalities)

∙ Population (P): Individuals with a confirmed ASD diagnosis or those undergoing evaluation or suspected of having ASD; additionally, general population samples assessed using screening or trait-based instruments.

∙ Exposure (E): Sex assigned at birth and/or gender; race/ethnicity; socioeconomic status; previous diagnoses (e.g., ADHD); social camouflaging or masking behaviours; systemic or institutional barriers.

∙ Comparison (C): Contrasting groups (e.g., girls vs. boys; AFAB vs. AMAB; non-Hispanic White individuals vs. racial/ethnic minorities; low vs. higher socioeconomic status; individuals with vs. without a prior ADHD diagnosis).

∙ Outcomes (O): Age at diagnosis, diagnostic delay and waiting times, prevalence, access to diagnostic assessment, screening and diagnostic performance, psychometric properties of assessment tools, and qualitative experiences of bias or diagnostic barriers.

Complementary PICO Framework (Diagnostic Tools and Technological Approaches)

∙ Population (P): Children and adolescents (particularly ages 0–6 and 6–18) and/or individuals referred for diagnostic evaluation due to suspected ASD.

∙ Intervention (I): Screening or diagnostic instruments and technological approaches (e.g., SCQ, ADOS, Canvas Dx, point-of-view technologies combined with computer vision, machine learning applied to parental reports or behavioural data, eye-tracking paradigms).

∙ Comparison (C): Standard clinical assessment, non-autistic control groups, or comparisons between subgroups defined by sex/gender or diagnostic status.

∙ Outcomes (O): Diagnostic accuracy indices (sensitivity, specificity, positive and negative predictive values), differential performance by sex/gender, and indicators of ecological validity or utility for early identification.

The combined use of PECO and PICO frameworks facilitated a systematic evaluation tailored to the methodological diversity of the included studies, allowing for the assessment of both population-level diagnostic inequalities and sex- or gender-related differences in the performance of diagnostic tools.

In the initial search phase, 142 records were identified across the selected databases. After removing 11 duplicates, 131 unique articles remained for screening. Title and abstract screening resulted in the exclusion of 16 records that did not meet the inclusion criteria, leaving 115 articles for full-text assessment.

During full-text review, 49 articles were excluded because they were systematic or narrative reviews. Of the remaining 66 studies, 21 did not address diagnostic inequalities related to sex or gender; 6 did not report relevant diagnostic or screening outcomes (e.g., age at diagnosis or instrument performance) or lacked comparative analyses; and 13 were excluded for not meeting the predefined publication time frame. As a result of this process, a final sample of 26 studies was included in the review (see Fig. 1).

### 2.4 Methodological Quality Assessment

The methodological quality of the included studies was assessed using the Critical Review Form for Quantitative Studies (CRF-QS) developed by Law *et al*. (1998) [7]. Given the marked heterogeneity of study designs included in this review, the CRF-QS was applied by prioritising criteria common to quantitative research, including clarity of objectives, adequacy of sample description, validity and reliability of measures, appropriateness of data analysis, and consistency between results and conclusions.

Although tools such as the Newcastle–Ottawa Scale or ROBINS-I are frequently employed in observational research, the CRF-QS was selected due to its flexibility and applicability across diverse quantitative designs. This choice may limit direct comparability with other systematic reviews and is therefore acknowledged as a methodological limitation. For qualitative studies, methodological appraisal focused on transparency in sampling strategies, data collection procedures, analytic processes, and reflexivity, with limitations in cross-study comparability explicitly reported.

The CRF-QS consists of 19 criteria scored dichotomously (1 = criterion met; 0 = criterion not met). Studies were classified according to total score using the categories proposed by Law *et al*. [7]: ≤11 = poor; 12–13 = acceptable; 14–15 = good; 16–17 = very good; and ≥18 = excellent [7].

CRF-QS scores across the included studies ranged from 13 to 18 points, with a mean score of 15.7, indicating overall good to very good methodological quality. Specifically, six studies were rated as excellent (≥18), nine as very good (16–17), seven as good (14–15), and four as acceptable (12–13). No studies were classified as having poor methodological quality.

#### Risk of Bias Assessment

Assessment of risk of bias is a core component of systematic reviews, as methodological bias can substantially distort findings and compromise the validity of conclusions. Risk of bias was evaluated using the classic Cochrane domains, adapted for non-randomised studies and interpreted in accordance with the specific characteristics of the included designs (observational, psychometric, quasi-experimental, and qualitative). The domains were operationalised as follows: (a) Selection bias, referring to sample representativeness and recruitment procedures (e.g., population-based versus clinical samples, inclusion and exclusion criteria, and group comparability); (b) Performance bias, defined as the degree of standardisation in data collection procedures and the consistency of assessment conditions across participants; (c) Detection bias, relating to the quality and consistency of outcome measurement (e.g., use of validated instruments, uniform measurement across groups, and evaluator blinding where applicable); (d) Attrition bias, associated with participant loss, follow-up completeness, and analytical handling of missing data, particularly in longitudinal designs; and (e) Reporting bias, concerning selective outcome reporting and transparency in the presentation of results.

Each study was classified into one of three risk-of-bias categories: (1) low risk, when methodological characteristics reduced the likelihood of systematic distortion; (2) uncertain risk, when available information was insufficient to allow a clear judgement; or (3) high risk, when evident methodological limitations were present (e.g., highly biased sampling, lack of standardisation, non-comparable measurements across groups, or substantial uncontrolled attrition).

A synthesis of risk of bias was prepared for each study according to its design, with brief justifications provided for each domain. Overall, 15 studies were judged to present a low risk of bias, nine an uncertain risk, and two a high risk of bias, primarily due to limitations in sample representativeness, small sample sizes, or exclusive reliance on self-reported data.

Both methodological quality appraisal and risk-of-bias assessment were conducted independently by the review author, using informed expert judgement based on the information reported in the original publications. Table 2 summarises the methodological quality (CRF-QS scores) and risk-of-bias assessments for all included studies [8, 9, 10, 11, 12, 13, 14, 15, 16, 17, 18, 19, 20, 21, 22, 23, 24, 25, 26, 27, 28, 29, 30, 31, 32, 33].

**Table 2.** **Assessment of study quality**.

Study	Design	CRF-QS	Quality	Risk of bias	Justification
Brian *et al*. (2016) [8]	Prospective longitudinal	17	Very good	Low	Blinded diagnosis, longitudinal follow-up, validated measures
Duvekot *et al*. (2017) [9]	Multicentre observational	16	Very good	Low	Clear sex-based comparisons, standardised instruments
Friedman *et al*. (2024) [10]	Qualitative (IPA)	14	Good	Uncertain	Small sample, but rigorous and transparent analysis
Goldblum *et al*. (2024) [11]	Population-based observational	17	Very good	Low	Large national sample, robust intersectional analysis
Hegemann *et al*. (2024) [12]	Population psychometric	18	Excellent	Low	Large N, EFA/CFA, measurement invariance testing
Kayış *et al*. (2026) [13]	Experimental	15	Good	Uncertain	Small sample, high precision but limited validation
Kentrou *et al*. (2019) [14]	Retrospective observational	15	Good	Uncertain	Retrospective clinical records, potential recall bias
Kniola *et al*. (2026) [15]	Cross-sectional observational	16	Very good	Low	Large SPARK cohort, clear predictive analyses
Levante *et al*. (2025) [16]	Cross-sectional	14	Good	Uncertain	Parent self-reports, no diagnostic confirmation
McKinney *et al*. (2024) [17]	Cross-sectional observational	15	Good	Uncertain	Self-reported camouflaging, moderate sample size
Morris & Campbell (2025) [18]	Population-based	16	Very good	Low	National survey, analyses by SES and gender
Parish-Morris *et al*. (2019) [19]	Cross-sectional	15	Good	Uncertain	Reliance on traditional ADOS
Peterson *et al*. (2024) [20]	Retrospective	14	Good	Uncertain	Small and gender-imbalanced sample
Probol & Mieskes (2025) [21]	Exploratory observational	13	Acceptable	High	Very small sample, social media recruitment
Rea *et al*. (2025) [22]	Cross-sectional	15	Good	Uncertain	Urban clinics, limited generalisability
Román-Urrestarazu *et al*. (2024) [23]	Population-based	18	Excellent	Low	National registries, robust methodology
Rutherford *et al*. (2018) [24]	Quasi-experimental pre–post	16	Very good	Low	Objective indicators, structural service improvement
Rutherford *et al*. (2016) [25]	Retrospective	15	Good	Uncertain	Real-world service data, referral bias
Salomon *et al*. (2025) [26]	Post-authorisation AI study	16	Very good	Low	Clear metrics, large sample size
Smith *et al*. (2024) [27]	Observational with mediation analysis	16	Very good	Low	Well-specified models, partial replication
Surgent *et al*. (2025) [28]	Longitudinal neuroimaging	17	Very good	Low	Longitudinal design, objective measures
Thomas *et al*. (2012) [29]	Epidemiological	15	Good	Uncertain	Older registry-based data
Tien *et al*. (2025) [30]	Qualitative	15	Good	Uncertain	Purposive sampling, strong theoretical rigour
Viktorsson *et al*. (2024) [31]	Longitudinal eye-tracking	17	Very good	Low	Early objective measures
Wieckowski *et al*. (2025) [32]	Observational	15	Good	Uncertain	Follow-up attrition
Zahorodny *et al*. (2025) [33]	Population surveillance	16	Very good	Low	Multiple registries, active surveillance

IPA, interpretative phenomenological analysis; EFA, exploratory factor analysis; CFA, confirmatory factor analysis; SPARK: simons foundation powering autism research for knowledge; SES, socioeconomic status; ADOS, autism diagnostic observation schedule.

Data extraction was conducted in accordance with the PECO framework for diagnostic inequalities and, where applicable, a complementary PICO framework for studies focusing on technological or instrument-based approaches (e.g., screening tools, diagnostic assessments, or artificial intelligence–based systems). For each included study, the following information was systematically extracted: sample characteristics (sample size, age range, and recruitment context); exposure or variables of interest (sex assigned at birth and/or gender, race/ethnicity, socioeconomic status, prior diagnoses such as attention-deficit/hyperactivity disorder, and the presence of social camouflaging or masking behaviours); comparator groups; primary outcomes; and measurement methods.

To facilitate structured synthesis, study findings were organised into five thematic domains: (1) prevalence and epidemiological patterns; (2) age at diagnosis, diagnostic delay, and waiting times; (3) performance of screening and diagnostic instruments, including subgroup-specific biases; (4) psychometric properties and evidence of measurement invariance; and (5) qualitative evidence addressing diagnostic barriers, gender bias, and lived experiences within the diagnostic process.

## 3. Results

Results are presented according to the thematic domains identified through the review process. Table 3 (Ref. [8, 9, 10, 11, 12, 13, 14, 15, 16, 17, 18, 19, 20, 21, 22, 23, 24, 25, 26, 27, 28, 29, 30, 31, 32, 33]) provides an overview of the studies included in the synthesis.

**Table 3.** **Characteristics of the selected studies**.

Reference	Objective	Design	Sample (n)	Instruments	Key findings
Brian *et al*. (2016) [8]	To examine diagnostic stability and change in ASD from age 3 to middle childhood in a cohort of high-risk younger siblings.	Prospective longitudinal study with blinded best-estimate clinical diagnosis.	N = 67 high-risk siblings (mean age at follow-up: 9.5 years).	Blinded clinical evaluation using best-estimate diagnosis; standardised measures of autistic symptoms, receptive language, and cognitive functioning.	High overall diagnostic stability between age 3 and middle childhood (89.6%; κ = 0.76). Of those diagnosed with ASD at age 3, 94.4% retained the diagnosis. However, six children (∼9%) were diagnosed later after initially being classified as non-ASD. These cases showed lower autistic symptom severity and better receptive language at baseline, and higher cognitive abilities at follow-up. Findings highlight that subtle profiles may emerge over time, supporting the need for longitudinal follow-up, particularly in high-risk populations.
Duvekot *et al*. (2017) [9]	To analyse whether behavioural characteristics differentially influence the likelihood of receiving an ASD diagnosis by sex in clinically referred children.	Multicentre observational study with comparative analyses and logistic regression.	N = 231 referred children (130 ASD: 106 boys, 24 girls; 101 non-ASD: 61 boys, 40 girls), aged 2.5–10 years.	Developmental, Dimensional and Diagnostic Interview (short version); ADOS; CBCL; intelligence tests (WISC, WPPSI, Bayley).	Parent-reported restricted and repetitive behaviours were less predictive of ASD diagnosis in girls than in boys (interaction OR = 0.41). Emotional and behavioural problems increased diagnostic likelihood more strongly in girls (interaction OR = 2.44). No sex differences were found for global autistic impairment, sensory symptoms, or cognitive functioning. Results provide evidence of gender bias in diagnostic processes and help explain the under-identification of ASD in girls in clinical settings.
Friedman *et al*. (2024) [10]	To explore the lived experiences of women and gender-diverse individuals who self-identify as autistic without a formal diagnosis, and how autistic identity is constructed outside the clinical diagnostic system.	Qualitative study using Interpretative Phenomenological Analysis (IPA).	n = 6 adults (18–69 years): 4 women, 1 non-binary individual, and 1 genderfluid/genderqueer individual.	Online semi-structured individual interviews; analysis via IPA.	Three main themes emerged: (1) autistic self-discovery, facilitating self-acceptance and positive identity reconstruction; (2) living without diagnosis, characterised by masking, gender bias, lack of formal support, and self-accommodations; and (3) self-diagnosis and doubt, shaped by external invalidation and challenges to the medical model. Self-diagnosis was described as an empowering pathway to constructing a positive autistic identity outside deficit-based paradigms, particularly in the context of access barriers and gender bias.
Goldblum *et al*. (2024) [11]	To analyse how sex assigned at birth, race, and ethnicity interact to predict autism prevalence and age at diagnosis.	Observational study with secondary analyses; hierarchical linear regression (intersectional approach).	NSCH 2016–2021 (national population-based sample; estimated prevalence 1 in 38).	National Survey of Children’s Health (2016–2021); caregiver-reported age at diagnosis and demographic variables.	Estimated prevalence was 1 in 38, with a male-to-female ratio of 3.8:1. Diagnostic delays were observed among some girls/women, particularly in non-Hispanic subgroups. Overall, racially and ethnically minoritised children received diagnoses earlier than non-Hispanic White children, regardless of sex. Findings suggest that diagnostic delay in females is strongly associated with ethnicity and that earlier identification in minority groups may reflect phenotypic differences or alternative identification pathways.
Hegemann *et al*. (2024) [12]	To examine the psychometric properties of the Social Communication Questionnaire (SCQ) in the general population, assessing factorial structure and measurement invariance by sex and autism diagnostic status.	Population-based psychometric study with exploratory and confirmatory factor analysis and invariance testing.	EFA: 21,775 children; CFA: 21,674 children; autistic subgroup: 636 children.	SCQ (current version) completed at age 8; national autism registry data; Norwegian Mother, Father and Child Cohort Study (MoBa).	A five-factor model provided the best fit in both autistic and non-autistic children, though qualitative differences in factor meaning were observed between groups. The model demonstrated general consistency between boys and girls, supporting sex invariance in the general population. Authors caution against uncritical use of the SCQ as a trait measure in population samples and recommend further invariance testing of screening tools.
Kayış *et al*. (2026) [13]	To develop an objective and ecologically valid method for autism diagnosis using point-of-view (POV) glasses and computerised analysis.	Experimental study.	56 children aged 17–36 months (29 autistic, 27 non-autistic controls).	POV glasses; OpenFace 2.0; MediaPipe for analysis of eye contact, social smiling, name response, and head movement.	Autistic children showed reduced eye contact, shorter eye-contact duration, fewer social smiles, fewer name responses, longer response latencies, and shorter responses. An AdaBoost model achieved 91.07% accuracy, with sensitivity of 89.65%, specificity of 92.59%, and precision of 92.85%.
Kentrou *et al*. (2019) [14]	To examine whether a prior ADHD diagnosis is associated with delayed ASD diagnosis and to explore sex differences in this delay.	Comparative observational study (retrospective analysis of age at diagnosis).	2212 participants (1009 adults; 1126 children); 770 (35.3%) were women.	Structured clinical interviews and review of diagnostic history (age at ADHD and ASD diagnosis).	Children and adolescents with prior ADHD received an ASD diagnosis on average 1.8 years later than those without ADHD. The delay was greater in girls (2.6 years) than in boys (1.5 years). No significant sex differences were observed in adults. Findings suggest that symptom overlap between ADHD and ASD may mask autistic features, particularly in girls, delaying recognition and diagnosis.
Kniola *et al*. (2026) [15]	To examine how developmental milestones, demographic variables, and emotional/behavioural functioning predict whethe	Cross-sectional observational study with comparative analyses and predictive models within a national cohort.	5946 autistic girls and women with spoken language (SPARK). Groups: meeting the SCQ cut-off (n = 5186) vs. not meeting the cut-off (n = 760).	Social Communication Questionnaire (SCQ); Child Behaviour Checklist (CBCL); parent-reported developmental milestones and sociodemographic variables (parental education, race).	Girls with delays in motor milestones and toilet training, as well as those with higher parental education levels, were more likely to exceed the SCQ cut-off. Clinical scores on Thought Problems and Attention Problems (CBCL) predicted a positive screening result. Race, withdrawal/depression, and social problems were not associated with screening status. The findings suggest that autistic girls may require more evident symptoms or more pronounced developmental delays to be identified through screening, indicating a potential gender bias in detection and possible barriers to early access to intervention.
Levante *et al*. (2025) [16]	To investigate the association between autistic traits, internalising and externalising traits, dysregulation, and competence in children aged 18–36 months	Cross-sectional study using parent-report questionnaires.	361 children aged 18–36 months, with information provided by their mothers.	Online questionnaires (Q-CHAT and ITSEA) administered to mothers of children aged 18–36 months.	Preliminary results showed that autistic traits, externalising traits, and dysregulation were more prevalent in boys than in girls, whereas girls showed higher competence. Autistic traits were positively associated with internalising traits, externalising traits, and dysregulation, and negatively associated with competence. In addition, older children showed fewer autistic traits and higher competence than younger children.
McKinney *et al*. (2024) [17]	To examine whether social camouflaging is already present during the transition to adolescence (11–14 years), to describe its components (masking, compensation, and assimilation), to examine the effect of age, and to explore its relationship with anxiety and depression in neurodivergent and neurotypical girls.	Cross-sectional observational study with a participatory and transdiagnostic approach, including comparative analyses and hierarchical regression models.	119 girls (70 neurodivergent with diagnoses of autism, ADHD and/or DCD; 49 neurotypical), aged 11–14 years (M ≈ 12 years).	Camouflaging Autistic Traits Questionnaire – Adapted (CAT-Q-A); Revised Child Anxiety and Depression Scale (RCADS); Anxiety Scale for Children – ASD (ASC-ASD); parent-report questionnaires (SRS-2, Conners-3, DCDQ).	Neurodivergent and neurotypical girls showed similar levels of camouflaging in masking and compensation, but neurodivergent girls scored significantly higher in assimilation (feeling as though they were “acting or pretending” to fit in). Age predicted higher levels of camouflaging. Camouflaging strongly predicted higher levels of anxiety and depression in both groups, eliminating the effect of neurotype in the models. These findings indicate that camouflaging emerges early and constitutes a key risk factor for mental health, particularly relevant for understanding psychological distress in neurodivergent girls.
Morris & Campbell (2025) [18]	To investigate disparities in autism diagnosis between 2016 and 2021 in relation to race, gender, and socioeconomic status (SES), and to determine whether these factors contribute to diagnostic delays.	Observational study based on survey data.	225,443 participants aged 3–17 years from the US National Survey of Children’s Health (2016–2021).	US Census surveys, with statistical analysis of diagnostic rates and age of identification.	2.5% of children were diagnosed with autism. Boys were diagnosed more frequently than girls (4.3% vs. 1.2%). Girls were diagnosed later (5.6 years vs. 4.9 years). Diagnostic rates across racial groups were similar, although White children were diagnosed later than Black, Hispanic, and other racial groups. Children living in poverty had lower diagnostic rates (2.7% vs. 4.2% among those not living in poverty).
Parish-Morris *et al*. (2019) [19]	To explore differences in the measurement of autism symptoms in young girls and boys and how this influences early identification.	Cross-sectional study.	4550 children (20–40 months) with either high or low familial risk of developing autism.	Autism Diagnostic Observation Schedule (ADOS).	The study found that the ADOS does not measure autism-related challenges equally effectively in girls and boys. Autistic girls tend to be diagnosed later because current instruments do not adequately capture their symptoms, particularly as many tools were originally developed primarily using male samples.
Peterson *et al*. (2024) [20]	To evaluate gender differences in the effects of Applied Behaviour Analysis (ABA) therapy on behavioural goals and characteristics in individuals with ASD.	Retrospective study.	100 participants (89 children and 4 adults).	Functional analysis, discrete trial training, massed training, naturalistic training, and Catalyst software.	No significant gender differences were found across the nine variables examined (percentage of mastered goals, days of instruction, etc.). This suggests that ABA-based interventions may be equally effective for both genders.
Probol & Mieskes (2025) [21]	To create a reproducible dataset of speech data and transcripts from women and men on the autism spectrum.	Observational study using social media data collection.	14 autistic women and 4 autistic men.	Collection of videos and speech transcripts using automatic transcription tools (OpenAI Whisper).	A total of 2641 videos from autistic women and 765 from autistic men were collected. The average video length was longer for men (8:26) than for women (6:25). The research showed that most autistic creators use social media to share autism-related experiences, and speech anomalies such as echolalia and repetitions were annotated.
Rea *et al*. (2025) [22]	To compare a sample of paediatric patients diagnosed with ASD in primary care with the general clinic population and to analyse child and caregiver characteristics associated with age at diagnosis.	Cross-sectional study.	Patients diagnosed with ASD between March 2018 and February 2022 from two urban primary care clinics.	Medical record review, χ^2^ analysis, and bivariate and multivariate linear regression.	Patients diagnosed with ASD were more likely to be male, Hispanic, publicly insured, and medically complex than the general clinic population. Higher maternal education was associated with the ASD group. Variables associated with earlier diagnosis included connection to early intervention services, higher M-CHAT scores, greater continuity of care, and having private insurance. No significant associations were found with race/ethnicity or the Social Vulnerability Index.
Román-Urrestarazu *et al*. (2024) [23]	To estimate the school-age prevalence of ASD in Chile using health records and Bayesian analysis, and to examine unmet special education needs among children aged 6–18 years.	Observational study using Bayesian analysis of electronic health records and school data.	3 million school-age children (6–18 years).	Health Service (SSAS) and Bayesian modelling to estimate national and regional prevalence using Electronic health records from the Araucanía Sur	The age- and sex-adjusted national school prevalence was 0.46% (95% CI: 0.46–0.47%). The adjusted clinical prevalence in SSAS was 1.22% (95% CI: 1.16–1.28%). Bayesian projections estimated a national prevalence of 1.31% (95% CI: 1.25–1.38%). Disparities were observed by sex, ethnicity, health services, and rurality. Boys were six times more likely to be diagnosed than girls (OR 6.10, 95% CI: 5.82–6.41).
Rutherford *et al*. (2018) [24]	To evaluate the impact of a healthcare service improvement programme on reducing waiting times and improving the quality of the ASD diagnostic process in children, as well as its effect on the identification of girls.	Quasi-experimental pre–post study of clinical service improvement.	Children referred for ASD diagnostic assessment in a paediatric clinical service (pre–post service improvement cohort).	Clinical process indicators were reviewed (waiting time from referral to first appointment and to diagnostic feedback), with pre–post statistical analysis; implementation of evidence-based diagnostic pathways, systematic collection of clinical data, and professional training.	Significant reductions in waiting times were observed: from 14.2 to 10.4 weeks between referral and first appointment, and from 270 to 122.5 days between referral and diagnostic feedback. After service improvement, the proportion of girls identified increased, with the male-to-female ratio decreasing from 5.6:1 to 2.7:1, suggesting that structural improvements in diagnostic services may reduce gender bias. The findings support the value of efficient, standardised, and well-trained diagnostic models to improve access and equity.
Rutherford *et al*. (2016) [25]	To analyse gender ratios, age at referral and diagnosis, and the duration of the diagnostic evaluation in children and adults with ASD in real-world clinical services.	Retrospective observational study based on medical record review.	N = 150 children and adults recently diagnosed with ASD.	Review of clinical notes and diagnostic records from ASD assessment services.	The gender ratio was lower than expected and decreased with age, suggesting underdiagnosis in females during earlier stages. Girls were referred and diagnosed significantly later than boys. No differences were found in the duration of the diagnostic assessment between sexes, indicating that delays in females occur before referral rather than during the diagnostic evaluation process. The findings support the hypothesis of delayed recognition of ASD in girls and highlight the need to improve early detection.
Salomon *et al*. (2025) [26]	To evaluate the real-world performance of Canvas Dx, an artificial intelligence-based diagnostic system for detecting autism in children.	Post-authorisation aggregated data analysis study.	254 children with suspected developmental delay.	Canvas Dx (parent app, behavioural questionnaires, child videos, and clinician web portal).	The device showed a negative predictive value (NPV) of 97.6% and a positive predictive value (PPV) of 92.4%. Determinate results were obtained in 63% of cases. Sensitivity was 99.1% and specificity was 81.6%. The findings indicated that Canvas Dx enabled an autism diagnosis more than two years earlier than the current average age of diagnosis. Performance was similar to previous clinical trials, with improvements in the rate of determinate results and PPV. No significant differences were observed by sex or age group, although children younger than 48 months showed a higher PPV
Smith *et al*. (2024) [27]	To analyse the relationships between age at autism diagnosis, sex assigned at birth, and symptoms of anxiety and depression, and to evaluate whether age at diagnosis mediates the effect of sex on psychopathology.	Observational study (secondary analysis) using regression-based mediation models in two samples (clinical and research).	Clinical sample: n = 1035 (22.9% AFAB). Research sample (sex-balanced): n = 128 (43% AFAB).	Age at diagnosis data and measures/questionnaires of anxiety and depression analysed using mediation models.	In both samples, older age at diagnosis predicted higher levels of anxiety and depression symptoms. Sex did not directly predict anxiety. In the clinical sample, individuals assigned female at birth (AFAB) were diagnosed later than those assigned male at birth (AMAB), and a significant indirect effect was observed: AFAB → later diagnosis → greater anxiety–depression symptoms. In the research sample, sex predicted depression only in that sample and the mediation by age at diagnosis was not replicated. These findings highlight the clinical importance of diagnostic timing, particularly for AFAB individuals.
Surgent *et al*. (2025) [28]	To investigate how autism diagnosis and sex assigned at birth influence the associations between the dorsal striatum and fine motor development in autistic and non-autistic children.	Longitudinal study.	356 children (234 autistic; 128 girls) at baseline and 195 children at follow-up (113 autistic; 76 girls).	Fine motor assessment (VABS-II) and magnetic resonance imaging (MRI; T1 and diffusion).	Significant associations were observed between fine motor abilities and putamen volumes, particularly in autistic children. In autistic girls, larger putamen volumes were associated with poorer fine motor skills, whereas the opposite pattern was observed in autistic boys. Associations were also observed with corticostriatal microstructure, particularly in autistic girls, where these measures predicted long-term fine motor development.
Thomas *et al*. (2012) [29]	To examine the association between socioeconomic status (SES) and ASD prevalence, as well as differences in age at diagnosis and access to professional evaluations.	Observational epidemiological study based on population records and multivariable analysis.	N = 586 children with ASD (aged 8 years).	Review of educational and medical records; US Census data (2000) on median household income; multivariable statistical análisis.	The prevalence ratio between the highest and lowest SES groups was 2.2 after adjustment for covariates. In higher SES areas, more professional evaluations were conducted and diagnoses were made earlier, although considerable overlap existed between SES levels. The results suggest that differences in prevalence partly reflect inequalities in access to diagnostic services rather than true differences in incidence.
	ASD prevalence was higher in areas with higher socioeconomic status (17.2/1000 in areas with income > $ 90,000 vs. 7.1/1000 in areas ≤ $ 30,000).				
Tien *et al*. (2025) [30]	To explore how the male autism stereotype and gender socialisation expectations influence the diagnostic trajectories of autistic adults, particularly among those not socialised as male, including clinical bias, family influences, and suppression/camouflaging strategies.	Qualitative study using interviews and reflexive thematic analysis informed by gender socialisation theories and feminist disability models	24 autistic adults: 14 genderqueer individuals, 8 women, and 2 men. Semi-structured interviews and reflexive thematic analysis.	Clinicians and family members tend to interpret symptoms differently in non-male individuals, contributing to delayed or missed diagnoses.	Themes showed that autism diagnosis is often based on an implicitly male representation of autism, which does not adequately capture the experiences of individuals socialised as women. Female socialisation promotes the suppression of autistic traits (e.g., camouflaging and behavioural adjustment), reducing clinical recognition and reinforcing diagnostic bias.
Viktorsson *et al*. (2024) [31]	To analyse the temporal dynamics of social gaze in 18-month-old children later diagnosed with autism when observing naturalistic social interactions between other children.	Prospective longitudinal eye-tracking study comparing groups based on diagnostic likelihood and later diagnosis.	N = 98 children: low risk (n = 22), high risk without later diagnosis (n = 60), and high risk with later autism diagnosis (n = 16).	Eye-tracking during observation of videos of naturalistic social interactions; diagnostic assessment at 36 months	Low-risk children showed the expected increase in gaze to the girl’s face following the request for the toy, reflecting sensitivity to social context. Children later diagnosed with autism showed reduced temporal allocation of gaze to the relevant face during this key social moment compared with the other groups. The results indicate early alterations in the temporal synchronisation of social attention rather than a global absence of social gaze.
Wieckowski *et al*. (2025) [32]	To identify factors predicting attendance at a diagnostic evaluation following a positive autism screening result.	Observational study across two large-scale screening studies.	895 children from primary care screening studies.	Autism screening questionnaires, family interviews, and follow-up of attendance at diagnostic evaluations.	Attendance at diagnostic evaluation varied significantly between the two studies, but no differences were found by sex, race, ethnicity, or maternal education. Age at screening was a significant predictor, with higher attendance when screening occurred at 18 months (57%) compared with 12 months (38%) or 15 months (30%). Children who completed the evaluation had higher screening scores than those who did not attend (t > 1.798, *p * < 0.038).
Zahorodny *et al*. (2025) [33]	To estimate ASD prevalence in 2006 in the metropolitan New Jersey area and examine changes in prevalence and demographic characteristics between 2002 and 2006.	Population-based epidemiological surveillance study.	Population cohorts of children born in 1994 (n = 28,936) and 1998 (n = 30,570). Total population monitored ≈59,500; ASD cases identified: n = 533.	Review of educational and healthcare records, population surveillance of ASD, and documented clinical diagnostic criteria.	ASD prevalence increased significantly from 10.6/1000 in 2002 to 17.4/1000 in 2006 (*p * < 0.001). The increase was observed across subtypes and demographic groups. The male-to-female ratio was close to 5:1, and prevalence was higher among White children than among other ethnic groups. The authors suggest that the increase may partly reflect improved detection and awareness, although other factors cannot be ruled out. The findings highlight the need for greater service resources.

ASD, autism spectrum disorder; CBCL, child behaviour checklist; ADHD, Attention-Deficit/Hyperactivity Disorder; Q-CHAT, quantitative checklist for autism in toddlers; ITSEA, infant-toddler social and emotional assessment; DCD, developmental coordination disorder; SRS-2, social responsiveness scale, second Edition; DCDQ, developmental coordination disorder questionnaire.

The 26 included studies provide converging evidence of diagnostic inequalities, alongside clear limitations in current instruments for capturing female presentations and profiles characterised by fewer externalising symptoms. Findings are synthesised below according to the predefined thematic blocks.

### 3.1 Prevalence and Epidemiology

Epidemiological and population-based studies consistently report a higher prevalence of ASD diagnoses in males, with boy-to-girl ratios ranging from 3.8:1 to 6:1 depending on the context and data source [8, 11, 17, 22, 33]. These disparities appear across population surveillance studies and analyses of national surveys and health registries.

However, several studies suggest that these ratios may partly reflect differences in access to services, detection practices, and diagnostic pathways rather than true differences in incidence. In this regard, Thomas *et al*. [29] and Morris and Campbell [18] showed that children from more advantaged socioeconomic backgrounds are more likely to receive a diagnosis and tend to be identified earlier, whereas poverty is associated with a lower probability of diagnosis [18, 27]. Similarly, Román-Urrestarazu *et al*. [23] reported disparities by sex, ethnicity, and rurality in a Latin American context, underscoring the structural dimension of diagnostic inequality

### 3.2 Age at Diagnosis, Diagnostic Delay, and Waiting Times

Studies examining age at diagnosis consistently identified significant diagnostic delays in girls and AFAB (assigned female at birth) individuals across both clinical and population-based samples [14, 18, 19, 21]. This delay is associated with more complex clinical trajectories and an increased subsequent burden of psychopathology.

Kentrou *et al*. [14] showed that a prior diagnosis of ADHD is associated with delayed ASD diagnosis, with a larger delay observed in girls—supporting the notion of diagnostic “masking” driven by comorbidity and symptom overlap. Likewise, Brian *et al*. [8] found that subtler profiles may not be detected in early childhood and may only become apparent through longitudinal follow-up, reinforcing the value of ongoing surveillance and repeated assessment, particularly in high-risk populations.

From a service-delivery perspective, Rutherford *et al*. [24] demonstrated that structural improvements to diagnostic pathways can substantially reduce waiting times and increase the identification of girls, suggesting that a meaningful proportion of the observed bias is modifiable at the level of healthcare systems and service organisation [19].

### 3.3 Performance of Screening and Diagnostic Instruments and Subgroup-Related Biases

A substantial body of evidence indicates that commonly used screening and diagnostic instruments are less sensitive to female autism presentations. Duvekot *et al*. [9] showed that restricted and repetitive behaviours are less predictive of an ASD diagnosis in girls, whereas emotional and behavioural difficulties exert a stronger influence on diagnostic likelihood in females. Similarly, Kniola *et al*. [15] found that girls require more pronounced developmental delays or a higher overall symptom burden in order to exceed established cut-off scores on the Social Communication Questionnaire (SCQ).

Studies focusing on direct observational assessment further reinforce these findings. Parish-Morris reported that the Autism Diagnostic Observation Schedule (ADOS) does not capture autism-related challenges with equal sensitivity in young girls, contributing to later identification and diagnosis [18]. In contrast, emerging technology-based approaches have shown promising results. Kayış *et al*. [13] and Salomon *et al*. [26] reported high diagnostic accuracy using artificial intelligence—based systems, with no significant differences in performance by sex, suggesting potential to mitigate human-related bias during the early stages of the diagnostic process [25].

### 3.4 Psychometric Properties and Measurement Invariance

Psychometric studies provided critical insights into the structural limitations of widely used instruments. Hegemann *et al*. [12] demonstrated that although the SCQ shows measurement invariance by sex at the population level, qualitative differences in factor structure emerge between children with and without an autism diagnosis. These findings raise concerns regarding the use of the SCQ as a continuous measure of autistic traits in general population samples [12].

Complementarily, Levante *et al*. [16] identified early sex-related differences in the expression of autistic, internalising, and externalising traits during early childhood, suggesting that developmental trajectories diverge prior to the typical age of clinical identification. Taken together, these results support the hypothesis that current diagnostic criteria and assessment tools are more closely aligned with externalising, male-typical phenotypes of autism [16].

### 3.5 Qualitative Evidence on Barriers, Biases, and Lived Experience

Qualitative studies provided in-depth insight into the mechanisms underlying diagnostic bias. Friedman *et al*. [10] and Tien *et al*. [30] described how the implicitly male stereotype of autism, together with gendered socialisation norms, encourages camouflaging and suppression of autistic traits. These processes hinder clinical recognition and promote self-diagnosis as an alternative means of identity validation and self-understanding [10, 29].

These findings are consistent with quantitative evidence on camouflaging during adolescence. McKinney *et al*. [17] showed that camouflaging behaviours emerge early and are strongly associated with increased anxiety and depressive symptoms, regardless of neurotype. Similarly, Smith *et al*. [27] demonstrated that later age at diagnosis mediates the relationship between being AFAB and greater anxiety–depressive symptomatology, highlighting the significant clinical impact of delayed diagnosis [26].

Finally, studies such as Wieckowski *et al*. [32] and Rea *et al*. [22] indicated that once initial barriers to access are overcome, there are no substantial sex differences in attendance at diagnostic evaluations or in the assessment process itself. This pattern reinforces the notion that gender bias primarily arises prior to referral and during early identification, rather than during the formal diagnostic assessment phase [21, 31].

## 4. Discussion

The findings of this systematic review indicate that gender bias in ASD diagnosis is a consistent and multifactorial phenomenon arising from the interaction between phenotypic characteristics, assessment practices, and broader contextual factors. Across the 26 studies reviewed, women and individuals assigned female at birth were found to present, on average, less externalising symptom profiles and higher levels of social camouflaging. These features substantially reduce the likelihood of identification through standard screening and diagnostic procedures.

These sex-related differences do not reflect a lower presence of autism in females, but rather highlight limitations in current diagnostic criteria and tools, which remain largely calibrated to male-typical presentations. As a result, females on the autism spectrum are more likely to experience diagnostic delay, under-identification, and more complex diagnostic trajectories, as well as a higher burden of mental health comorbidities, particularly anxiety and depression [34]. Given the well-established association between earlier diagnosis and improved access to support and outcomes, these disparities have important clinical and public health implications.

A central theme across the reviewed literature is the role of social camouflaging and less prototypical phenotypic profiles in shaping the detection of ASD in females [35, 36]. Evidence suggests that from early childhood, girls may engage in compensatory, masking, and social assimilation strategies that reduce the overt visibility of core autistic difficulties, particularly in structured assessment contexts. While these strategies may facilitate short-term social adaptation, they also contribute to reduced sensitivity of screening and diagnostic instruments that were predominantly developed and validated using male samples.

Accordingly, diagnostic delay should not be conceptualised solely as an individual-level phenomenon, but rather as the result of a systematic mismatch between female autism phenotypes and dominant diagnostic models (Ketelaars *et al*., 2017 [37]; Lewis *et al*., 2021 [38]). This misalignment underscores the need for more gender-sensitive diagnostic frameworks that account for variability in symptom expression, developmental trajectories, and contextual influences.

**Fig. 2.**
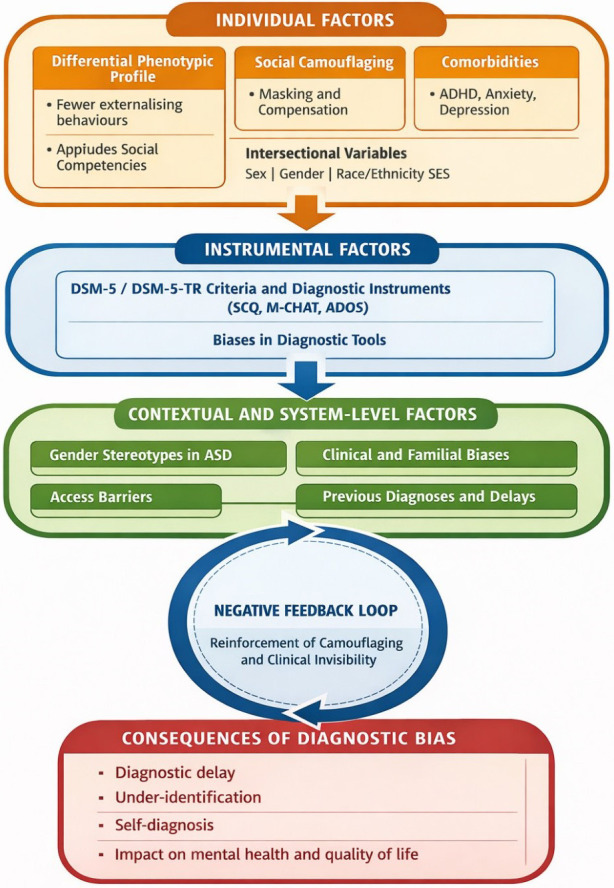
**Main mechanisms involved in gender bias in ASD diagnosis: individual, instrumental, and contextual factors across the diagnostic trajectory**.

To integrate these findings and provide a comprehensive explanatory framework, Fig. 2 presents a conceptual model synthesising the main mechanisms involved in gender bias in ASD diagnosis. This model incorporates individual, instrumental, and contextual factors operating across different stages of the diagnostic trajectory.

Based on this model, the results suggest that instrumental factors play a key role in perpetuating gender bias. Several studies show that the most widely used screening and diagnostic tools (e.g., SCQ, ADOS, M-CHAT) have limitations in detecting female autistic profiles, especially in girls and women with oral language and average or high cognitive ability [37, 38]. In this regard, psychometric studies indicate that, although some instruments maintain a stable factor structure by sex, their diagnostic performance depends on the presence of more visible or externalising symptoms, which places many girls below the clinical cut-off points [39]. This pattern reinforces the idea that the absence of functional invariance in clinical practice—beyond statistical invariance—contributes to the under-identification of ASD in women, especially when social camouflage reduces the observable expression of core symptoms.

Complementarily, contextual and diagnostic system factors emerge as critical modulators of the diagnostic trajectory. Qualitative and observational evidence points to how gender stereotypes, normative expectations, and implicit biases in both professionals and families tend to differentially interpret the same behaviours depending on whether the person is female or male [40]. Likewise, variables such as socioeconomic status, race/ethnicity, and the existence of previous diagnoses—particularly ADHD or internalising disorders—interact with gender to delay or divert the diagnostic process [41]. These data are consistent with an intersectional reading of diagnostic bias, in which inequalities are not distributed homogeneously but are intensified in certain subgroups of autistic women.

Finally, the longitudinal and mental health studies included in the review highlight the clinical and psychosocial consequences of these prolonged diagnostic trajectories. Delayed recognition of ASD is consistently associated with higher levels of anxiety, depression, and psychological distress, as well as greater reliance on self-diagnosis in adulthood as a strategy for understanding identity [37, 38]. As illustrated by the negative feedback loop in the conceptual model, clinical invisibility reinforces camouflage, which in turn perpetuates the lack of diagnostic recognition. Taken together, these results underscore the need to critically review current diagnostic models and move towards more gender-sensitive approaches that integrate diverse phenotypic profiles, multi-method assessment, and explicit clinical training in gender bias and intersectionality. This reinforces the need to develop diagnostic tools that are sensitive to phenotypic presentation, as proposed by Kirkovski *et al*. [41].

### Limitations and Future Lines of Research

This review has several limitations that should be considered when interpreting its findings. First, the evidence included was highly heterogeneous in terms of study designs (observational, psychometric, qualitative, and technological), populations, assessment instruments, and outcome measures. This heterogeneity limits direct comparability between studies and precludes quantitative synthesis or meta-analytic approaches.

Second, although the selection process was reported in accordance with the PRISMA framework, the review protocol was not prospectively registered. The absence of prior registration may increase the risk of bias related to a posteriori analytical decisions, such as adjustments to inclusion criteria or the thematic grouping of results.

Third, the bibliographic search was conducted in databases primarily focused on psychology and education (APA PsycInfo, ERIC, Dialnet, and PsicoDoc). Multidisciplinary and biomedical databases such as Scopus, Web of Science, or PubMed/MEDLINE were not included, which may have limited the identification of some international studies, particularly those originating from neurological or biomedical research contexts. As a result, the generalisability of the findings to other disciplinary and geographical settings may be partially constrained.

Furthermore, although systematic reviews are well suited to integrating evidence and identifying convergent patterns, other synthesis approaches could complement or extend the present findings. For example, meta-analyses—where sufficient homogeneity of outcomes exists—could provide pooled estimates of effect sizes for diagnostic delay or instrument performance. Scoping reviews might offer a broader mapping of emerging and grey literature, while realist or theory-driven reviews could help elucidate what works, for whom, and under which conditions in reducing diagnostic inequalities. Mixed-methods syntheses would also be valuable in more systematically integrating quantitative and qualitative evidence on camouflaging, systemic barriers, and mental health consequences.

From a clinical and public health perspective, these limitations reinforce the need to advance towards diagnostic approaches that are more sensitive to gender and intersectionality. This includes the use of longitudinal, multi-method, and contextually informed assessments, as well as targeted training for professionals in recognising implicit bias and phenotypic variability. There is also a clear need for the development and validation of diagnostic tools that more accurately capture female autism profiles, in order to reduce diagnostic delay and its associated emotional and psychosocial consequences. Addressing these methodological and structural limitations is essential to ensure equitable access to diagnosis, support, and early intervention, and to promote a more inclusive and accurate understanding of the autism spectrum.

It should be noted explicitly that this systematic review was not prospectively registered in PROSPERO or the Open Science Framework (OSF), which constitutes a methodological limitation. Nevertheless, the inclusion criteria, search strategy, and outcome domains were defined a priori and applied consistently throughout the review process.

## 5. Conclusion

In summary, the findings of this systematic review demonstrate that gender bias in ASD diagnosis is a structural and multifactorial phenomenon, sustained by the convergence of less prototypical phenotypic profiles, insufficiently sensitive assessment practices, and sociocultural contexts shaped by gendered expectations. Importantly, the reviewed evidence indicates that this bias does not reflect a lower prevalence of ASD in women, but rather the invisibilisation of certain forms of autistic expression within current diagnostic models, leading to later and more complex diagnostic trajectories [42].

With regard to the first objective, the literature consistently documents a persistent diagnostic disproportion by sex/gender, modulated by contextual factors and sources of identification, pointing to inequalities in detection and access to diagnostic services. In relation to the second objective, the evidence indicates a clear diagnostic delay in girls and AFAB individuals, particularly when comorbidities such as ADHD or internalising symptoms are present, or when profiles involve higher cognitive functioning and fewer externalising behaviours.

Addressing the third objective, the review shows that several widely used screening and diagnostic instruments display reduced sensitivity to female autism presentations, often placing girls below established cut-off thresholds despite clinically significant difficulties recognised by families or caregivers. In parallel, emerging technological tools show promise in reducing subjective bias, although further independent validation and equity-focused evaluation are required. Regarding the fourth objective, psychometric evidence highlights the urgent need to examine sex- and gender-related differences more systematically, as much of the existing literature remains based on predominantly male samples, reinforcing the conceptualisation of autism as a male-typical condition. Current research makes it clear that girls and women with ASD—particularly those at Level 1 and without intellectual disability—do exist, but remain under-represented and under-recognised within clinical and research frameworks.

Finally, in relation to the fifth objective, qualitative studies emphasise the role of social camouflaging, gender stereotypes, and systemic barriers in contributing to delayed or missed diagnoses, and link these processes to increased emotional distress. These findings underscore the necessity of an intersectional perspective in understanding diagnostic inequities. Diagnosis of a neurodevelopmental condition should extend beyond the assignment of a clinical label, serving instead as a gateway to timely intervention, support, and improved quality of life. The absence or delay of diagnosis places girls, adolescents, and women at increased vulnerability to mental health difficulties.

Taken together, these findings support the need to move towards multi-method, longitudinal, and gender-sensitive diagnostic approaches that incorporate explicit training in bias awareness, contextualised assessment, and tools adapted to phenotypic diversity. Such advances are essential to ensure equitable access to diagnosis and support, and to foster a more inclusive and accurate understanding of autism across the full spectrum of presentations.

## Availability of Data and Materials

The extracted data are available from the corresponding author upon reasonable request.

## Supplementary Material

Supplementary material associated with this article can be found, in the online version, at https://doi.org/10.31083/RN49367.
